# Treatment of Vaginitis

**Published:** 1855-09

**Authors:** 


Treatment of Vaginitis.—The *Union Medicale* of Jan. 18th, contains an interesting paper by MM. Bebquerel and Rodier, on the different modes of treatment employed in vaginitis, founded on observations made at the Hospital of Lourcine, at Paris. Although no description is given of the disease, we presume that most of the cases were those of acute and chronic *gonorrhœa*, as the hospital is designed exclusively for the treatment of the venereal diseases of women. The following applications were employed for a considerable length of time upon a number of patients. 1. A concentrated solution of nitrate of silver. 2. A more diluted solution of the same (16 parts of the salt to 120 of water.) 3. The solid nitrate of silver. 4. Tincture of iodine. 5. An ointment composed of lard and alum. 6. A concentrated solution of tannin. 7. Benzia, employed internally, as well as locally. Of all these applications the writers consider the concentrated solution of tannin (equal parts, by weight, of tannin and distilled water), applied directly upon the inflamed mucous membrane of the vagina, to be the best, as being the least painful, and least offensive. Of 28 cases treated in this way, all were cured, the average time being from 20 to 27 days, and the number of applications from 7 to 8. The tincture of iodine was found to be an excellent application for chronic and acute vaginal leucorrhœa, not accompanied by an inflammatory condition of the mucous membrane; requiring between twelve and thirteen days, and four or five applications.—*Boston Med. and Surg. Jour.*

